# Antithrombotic agents and effect on outcomes in ischemic stroke with atrial fibrillation and large artery atherosclerosis: a real-world study

**DOI:** 10.3389/fneur.2025.1576250

**Published:** 2025-06-09

**Authors:** Jilu Chen, Jianhua Cheng, Qiang Ye, Yuntao Liu, Yanlei Zhang, Zheng Zhang

**Affiliations:** ^1^The First School of Clinical Medicine, Wenzhou Medical University, Wenzhou, China; ^2^Department of Neurology, Wenzhou Medical University First Affiliated Hospital, Wenzhou, China

**Keywords:** acute ischemic stroke, atrial fibrillation, antithrombotic agents, anticoagulants, large artery atherosclerosis, real world, effect

## Abstract

**Introduction:**

The optimal antithrombotic regimen for preventing recurrent stroke in patients who experience ischemic stroke due to atrial fibrillation (AF) and atherosclerotic large-vessel stenosis remains unclear. The present study aimed to evaluate the effect of multiple antithrombotic therapies on outcomes after ischemic stroke due to ≥ 2 causes.

**Methods:**

Data from 632 patients at a single hospital, who experienced ischemic stroke due to AF and large-artery atherosclerosis. Patients were categorized into 3 groups according to antithrombotic therapy at discharge: antiplatelets (APT), oral anticoagulant(s) (OAC), and APT plus OAC. Study outcomes included recurrent ischemic stroke and composite outcomes for cardiovascular events, death and major bleeding. Propensity scores (PS) were used to balance APT and OAC groups.

**Results:**

Among 632 patients, 158 (25.0%) were treated with APT, 447 (70.7%) with OAC, and 27 (4.3%) with both APT and OAC. After applying PS, only OAC had a significant beneficial effect on the composite outcome (hazard ratio [HR] 0.41 [95% confidence interval (CI) 0.19–0.83]; *p* = 0.01) and death (HR 0.12 [95% CI 0.01–1.0]; *p* = 0.05). However, there was no significant difference in one-year recurrent stroke events or risk for bleeding between the APT and OAC groups. Further analysis of the relationship between the dose of OAC and outcome revealed no significant difference between reduced and standard doses of OAC.

**Conclusion:**

This study demonstrated that OAC monotherapy was associated with a lower risk for composite outcomes and death after ischemic stroke due to AF and atherosclerotic stenosis, although the OAC dose had no effect on clinical outcomes.

## 1 Introduction

Atrial fibrillation (AF) is a leading cause of cardioembolic stroke, which is often fatal or disabling. Long-term oral anticoagulant(s) (OAC) therapy is usually recommended to reduce the recurrence of embolic events in patients diagnosed AF (1–5). However, the development of acute ischemic stroke (AIS) in patients with AF undergoing OAC treatment has become an increasingly important issue in clinical practice. One of the reasons for this is AF-unrelated stroke, since it has been estimated that one-third of the patients with ischemic stroke and AF may also have concomitant large-artery atherosclerosis (LAA) or small-vessel disease (SVD) (6–9). Emerging evidence suggests that AF and atherosclerosis exhibit a bidirectional pathophysiological interplay beyond shared traditional risk factors. Mechanistically, atherosclerotic lesions induce atrial electrical remodeling, thereby facilitating AF initiation and perpetuation. Conversely, AF exacerbates atherosclerosis via two key pathways: endothelial dysfunction and systemic inflammation. This positive feedback mechanism creates a vicious cycle, wherein AF and atherosclerosis mutually reinforce disease progression (10). Notably, the coexistence of atherosclerosis and AF confers a synergistic elevation in thromboembolic risk (11). Due to the lack of randomized clinical trials, there are no guidelines for antithrombotic treatment in patients with AIS and simultaneous AF and LAA. There are broadly 3 alternative antithrombotic regimens: monotherapy with OAC, monotherapy with an antiplatelet (APT) agent and combined OAC and APT agent. The present real-world study aimed to investigate and compare the therapeutic efficacy of AIS, AF, and LAA in patients, who experienced recurrent ischemic stroke over a period of 1 year.

## 2 Materials and methods

### 2.1 Study population

This retrospective study enrolled consecutive patients diagnosed with AIS, nonvalvular atrial fibrillation (NVAF) and LAA between January 2017 and December 2022 at The First affiliated Hospital of Wenzhou Medical University, Wenzhou, China. Data from patients diagnosed with AIS (within 7 days of symptom onset) between January 2017 and December 2022 due to ≥ 2 potential causes (NVAF and LAA > 50% in the relevant intracranial or extracranial vessels) according to the Trial of Org 10172 in Acute Stroke Treatment (TOAST) classification (12), were included. After excluding non-atherosclerotic etiologies, intracranial large artery atherosclerosis (LAA) with ≥ 50% luminal stenosis was confirmed by digital subtraction angiography (DSA), computed tomography angiography (CTA), or magnetic resonance angiography (MRA), consistent with the diagnostic criteria for atherosclerotic stenosis in relevant intracranial arteries. Patients who met the following criteria were excluded: non-AF/LAA causes, incomplete imaging/ECG data, poor follow-up feasibility, treatment conflicts (anticoagulation intolerance, prior revascularization), vascular malformations, pregnancy, or ambiguous findings (stenosis <50%, unverified AF episodes). In addition, discharge medications prescribed after stroke, documented in hospital records, were also analyzed.

The data involved in this study are anonymous retrospective data, which are exempted from ethical review after being reviewed by the Ethics Review Committee of the First Affiliated Hospital of Wenzhou Medical University. The research strictly follows the principles of Helsinki Declaration to protect the privacy of subjects. Requirements for informed consent were waived by the Institutional Review Board of the First Affiliated Hospital of Wenzhou Medical University due to the retrospective design of the study and the use of anonymized data.

### 2.2 Baseline characteristics and clinical information

Baseline characteristics analyzed included the following: age, sex, vascular risk factors (hypertension [HT], diabetes mellitus [DM], dyslipidemia [DL], coronary artery disease [CAD], AF, previous stroke/transient ischemic attack [TIA], and smoking). Clinical information for acute stroke management included the following: initial National Institutes of Health Stroke Scale (NIHSS) score (13); stroke mechanisms; thrombolytic therapy including intravenous (IV) thrombolysis, and endovascular recanalization therapy (EVT); hemorrhage transformation after stroke; length of hospitalization; CHA_2_DS_2_-Vasc (2 points for history of stroke or age > 75 years, and 1 point each for congestive heart failure, HT, DM, vascular disease, age 65 to 74 years, and female sex) and HAS-BLED (Hypertension, Abnormal Renal/Liver Function, Stroke, Bleeding History or Predisposition, Labile International Normalized Ratio [INR], Elderly, Drugs/Alcohol Concomitantly) score after the index ischemic stroke were calculated (14, 15). Information regarding discharge medications, including APT agents, OACs (warfarin and direct OAC) or both, was also collected. Patients were categorized into 3 groups according to the prescribed antithrombotic therapy at discharge: APT agents only; OACs only; and APT agents plus OACs. The APT group was selected as the reference group because it was expected to be the least effective treatment option among the 3 regimens. The doses of non-vitamin K antagonist OACs (NOACs) were recorded because low dose NOACs may be associated with high crude adverse event rates, particularly in patients who should have received standard NOAC dosing. Low dose dabigatran was considered to be labeled for elderly patients (age ≥ 80 years), patients with moderate renal impairment (creatinine clearance, 30–49 mL/min), and those with concomitant use of interacting drugs (eg, verapamil). Low dose rivaroxaban was considered to be labeled for patients with moderate or severe renal impairment (creatinine clearance, 15–49 mL/min) (16).

### 2.3 Outcomes

The primary outcomes were composite outcomes, including recurrent ischemic stroke, intracerebral hemorrhage (ICH), myocardial infarction (MI), and gastrointestinal bleeding (GI) and all-cause death 1 year after the index stroke. Secondary outcomes were major bleeding including ICH and GI. Patient status was assessed by telephone interview(s) or clinic visits, when possible.

### 2.4 Statistical analysis

Baseline characteristics are expressed as percentage (%), means with standard deviation (SD), and median with interquartile range. Continuous variables were analyzed using the Kruskal–Wallis test or one-way analysis of variance, as appropriate. Pearson’s *χ*^2^ test or Fisher’s exact test were used to analyze categorical variables in the 3 groups. A 1:1 propensity score (PS) matching method was used to minimize the effect of confounding factors for treatment comparisons on outcomes. The PS for each treatment group was developed using a logistic regression model with all covariates in the baseline characteristics (17). After 1:1 PS matching, the balance of covariates between the APT and OAC groups was assessed according to the absolute standardized mean differences (ASMD), with ASMD ≤ 0.1 considered to be an acceptable range of difference and was well-balanced in covariates between groups. The effect of treatment on outcomes was also evaluated using a weighted Cox proportional hazards regression model with PS matching. The cumulative incidence of outcomes was estimated using the adjusted Kaplan–Meier method (18). Differences with *p* < 0.05 were considered to be statistically significant. All statistical analyses were performed by professional medical statisticians using SPSS version 22.0 (IBM Corp., Armonk, NY, USA).

## 3 Results

### 3.1 Baseline characteristics

The baseline characteristics of patients included in this study (*n* = 632) are summarized in Table 1. Of the 632 patients, 31.2% (*n* = 197) were female, with a median age of 77 years. OACs (70.7% [*n* = 447]) were the most frequently used antithrombotic treatment regimen, followed by APT agents (25.0% [*n* = 158]), and a combination of APT agents and OAC treatment (4.3% [*n* = 27]). Patients in the APT group were older and had higher CHA_2_DS_2_-VASc scores than those in the other groups. The APT group also had a higher hemorrhage transformation rate after ischemia and more deaths within 1 year after stroke compared to the other 2 groups (Table 1). More patients in the APT plus OAC group had a history of CAD and more than one-half of patients in this group were diagnosed cardioembolic stroke (Table 1). To adjust for imbalances in baseline characteristics between the APT and OAC treatment groups, PS matching was performed. Patients who received combined APT and OAC from the PS analysis were excluded due to the small sample size of this subgroup. After PS matching, the balance of baseline covariates between before and after PS in APT and OAC groups was acceptable (Supplementary material).

**Table 1:** Baseline characteristics of included patients.

Characteristics	Total (*n* = 632)	Antiplatelet (*n* = 158)	Anticoagulant (*n* = 447)	Anticoagulant and antiplatelet (*n* = 27)	*p* value
Age, median, [IQR]	77 [70, 83]	79 [73, 87]	76 [72, 82]	76 [71, 79]	0.002*
Female, *n* (%)	197 (31.2)	58 (36.7)	134 (29.9)	5 (18.5)	0.190
Hypertension, *n* (%)	524 (82.9)	140 (88.6)	360 (80.5)	24 (88.9)	0.179
Diabetes mellitus, *n* (%)	68 (10.8)	23 (14.6)	40 (8.9)	5 (18.5)	0.168
Dyslipidemia, *n* (%)	44 (7.0)	11 (6.9)	31 (6.9)	2 (7.4)	0.974
Coronary heart disease, *n* (%)	75 (11.9)	20 (12.6)	43 (9.6)	12 (44.4)	<0.001*
Coronary artery stent implantation, *n* (%)	21 (3.3)	6 (3.8)	10 (2.2)^#^	5 (18.5)	0.003*
History of AF, *n* (%)	277 (43.8)	62 (39.2)	199 (44.5)	16 (59.3)	0.195
Congestive heart failure	151 (23.9)	46 (29.1)	103 (23.0)	2 (7.7)	0.069
Previous stroke/TIA, *n* (%)	140 (22.2)	36 (22.7)	98 (21.9)	6 (22.2)	0.985
Artery stenosis					
Single intracranial artery	197 (31.2)	41 (25.9)	144 (32.2)	12 (44.4)	0.11
Single carotid artery	44 (6.9)	14 (8.8)	28 (6.3)	2 (7.4)	0.11
Multiple arteries	391 (61.9)	103 (65.8)	275 (61.5)	13 (48.1)	0.11
Kidney failure, *n* (%)	268 (42.4)	77 (48.7)	183 (40.9)	8 (29.6)	0.162
Peripheral artery disease, *n* (%)	43 (6.8)	12 (7.5)	31 (6.9)	0	0.144
Smoking, *n* (%)	186 (29.4)	35 (22.7)	139 (31)	12 (44.4)	0.092
NIHSS median, [IQR]	3.0 [1, 8]	4.0 [1, 10]	3.0 [1, 3]	3.0 [1, 8]	0.41
Systolic pressure median, [IQR]	147[132, 163]	153[137, 166]	146 [131, 163]	144 [122, 170]	0.183
IV Thrombolysis, *n* (%)	90 (14.2)	23 (15.1)	66 (14.7)	1 (3.7)	0.175
EVT, *n* (%)	8 (1.3)	2 (1.2)	6 (1.3)	0	0.702
Hemorrhage transformation	64 (10.1)	34 (21.5)	29 (6.4) ^#^	1 (3.7)	NA
GI	9 (1.4)	2 (1.2)	6 (1.3)	1 (3.7)	NA
Rivaroxaban		-	393 (88.9)	23 (85.2)	NA
Dabigatran		-	51 (11.1)	3 (7.4)	NA
warfarin	4 (0.6)	-	3 (0.7)	1 (3.7)	NA
TOAST classification					
Atherosclerosis, *n* (%)	96 (15.2)	44 (27.8)	43 (9.6) ^#^	9 (33.3)	<0.001*
Cardioembolic, *n* (%)	319 (50.5)	47 (29.7)	266 (59.5) ^#^	5 (18.5)	<0.001*
Lacunar, *n* (%)	10 (1.6)	6 (3.7)	4 (0.8)	0	0.134
Other, *n* (%)	207 (32.8)	61 (38.6)	134 (29.9) ^#^	13 (48.1)	0.098
CHA_2_DS_2_-VASc>2, *n* (%)	468 (74.1)	134 (84.8)	312 (69.8) ^#^	22 (81.5)	0.013^*^
HAS-BLED, mean (SD)	2.1 (1.1)	1.8 (1.2)	2.2 (1.1)	2.0 (0.9)	0.056
Hospitalization day, median, [IQR]	9 [7, 12]	9.0 [6, 12]	9.0 [7, 12]	7.00 [6, 11]	0.250
Composite vascular (%)	125 (19.8)	42 (26.6)	77 (17.2)	6 (22.2)	0.038*
Ischemic stroke recurrence (%)	69 (10.9)	18 (11.4)	47 (10.5)	4 (14.8)	0.78
Major bleeding (%)	21 (3.3)	8 (5.1)	12 (2.7)	1 (3.7)	0.38
Death (%)	35 (5.5)	16 (10.1)	18 (4.0) ^#^	1 (3.7)	0.02*

AF, atrial fibrillation; TIA, transient ischemic attack; Kidney failure creatinine clearance <50 mL/min; NIHSS, National Institute of Health stroke scale; IV, Thrombolysis Intravenous thrombolysis; EVT, endovascular treatment; GI, gastrointestinal bleeding; TOAST, Trial of Org 10172 in Acute Stroke Treatment; *p < 0.05 between three groups; ^#^ *p* < 0.05 between antiplatelet and anticoagulant group.

### 3.2 Outcome

In the crude outcome analysis, the one-year composite outcome occurred in 19.8% of patients, including 10.9% with recurrent ischemic stroke and 3.3% with major bleeding (Table 1). Thirty-five (5.5%) patients died within first year, including 17 from major bleeding, 8 due to recurrent stroke and 10 cases of infection. A total of 105 (40.4%) patients with normal kidney function received a reduced, off-label dose of rivaroxaban.

**Figure 1:**
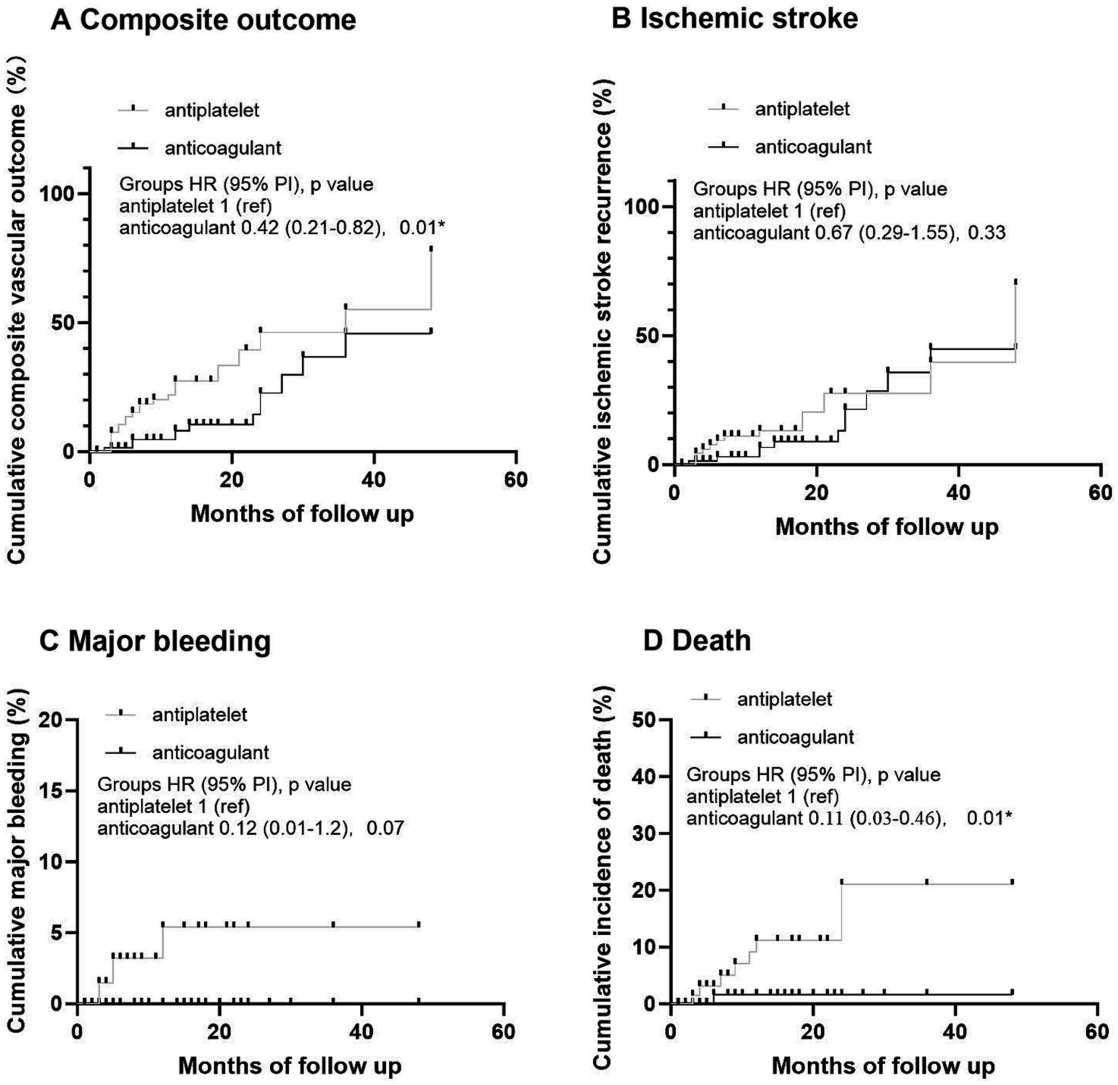
Kaplan–Meier analysis of primary and secondary outcomes in antiplatelet and anticoagulant groups. **(A)** Cumulative composite vascular outcome. **(B)** Cumulative ischemic stroke recurrence. **(C)** Cumulative major bleeding. **(D)** Cumulative incidence of death.

In the adjusted Cox proportional hazards regression analysis before adjustment after PS matching, compared with the APT group as a reference, the OAC group exhibited a lower risk for the composite outcome (hazard ratio [HR] 0.28 [95% CI 0.1–0.8]; *p* = 0.02). The one-year risks for recurrent ischemic stroke and major bleeding rates were similar among the groups (Table 2). After adjustment for age, PS matching, hemorrhage transformation after stroke, TOAST classification, and CHA_2_DS_2_-VASc scores, the difference between the APT and OAC groups remained significant for the composite outcome (weighted HR 0.3741 [95% CI 0.19–0.83]; *p* = 0.01). For secondary outcomes, mortality at 1 year was lower in the OAC groups before and after adjustment (HR 0.11 [95% CI 0.01–0.92], *p* = 0.04; HR 0.12 [95% CI 0.01–1.0], *p* = 0.05) (Table 2; Figure 1).

**Table 2:** Primary and secondary outcomes in antiplatelet and anticoagulant groups after PS matching.

Outcomes	No. of events	Unadjusted HR (95% CI)	*p* value	Adjusted HR (95% CI)	*p* value
Composite vascular	34 (24.3)				
APT	22 (31.4)	1 (Ref)	1 (Ref)	1 (Ref)	1 (Ref)
OAC	12 (17.1)	0.28 (0.1–0.8)	0.02*	0.41 (0.19–0.83)	0.01*
Ischemic stroke recurrence	23 (16.4)				
APT	12 (17.1)	1 (Ref)	1 (Ref)	1 (Ref)	1 (Ref)
OAC	11 (15.7)	0.38 (0.12–1.12)	0.08	0.67 (0.29–1.53)	0.34
Major bleeding	3 (2.2)				
APT	3 (4.3)	1 (Ref)	1 (Ref)	1 (Ref)	1 (Ref)
OAC	0 (0)	0.01 (0.00–143.60)	0.34	0 (0–9.7)	0.96
Death	8 (5.7)				
APT	7 (10.0)	1 (Ref)	1 (Ref)	1 (Ref)	1 (Ref)
OAC	1 (1.4)	0.11 (0.01–0.92)	0.04*	0.12 (0.01–1.0)	0.05*

PS, propensity score; APT, Antiplatelet; OAC, Oral anticoagulant; Adjusted for age, history of hypertension, congestive heart failure, kidney failure, CHA2DS2-VASc and HAS-BLED score. *p < 0.05.

**Figure 2:**
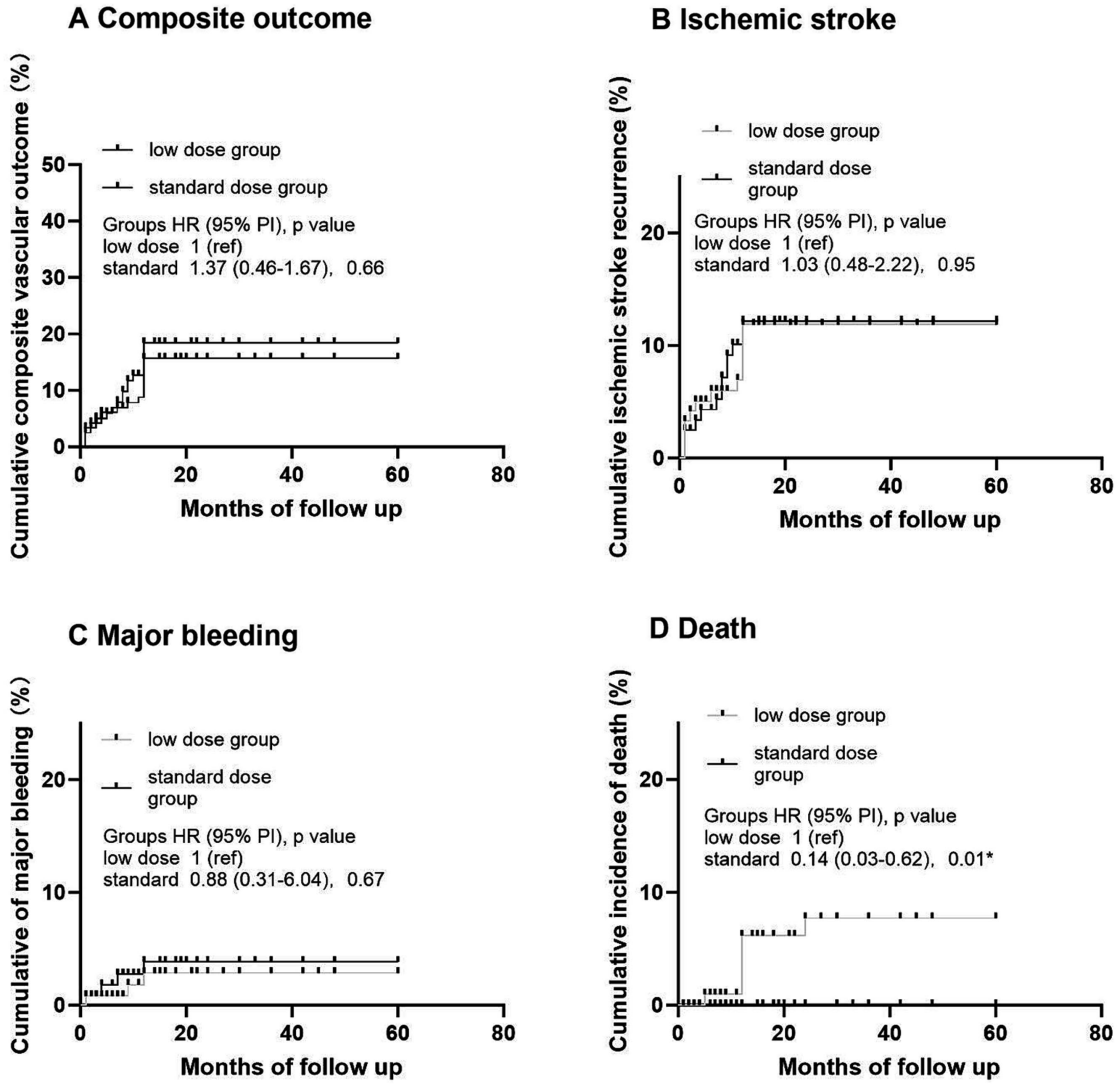
Kaplan–Meier analysis of primary and secondary outcomes in different dose of rivaroxaban. **(A)** Cumulative composite vascular outcome. **(B)** Cumulative ischemic stroke recurrence. **(C)** Cumulative major bleeding. **(D)** Cumulative incidence of death.

In OAC group, 393 (88.9%) patients were prescribed rivaroxaban, 51 (11.1%) were prescribed dabigatran, and 3 (0.7%) received warfarin therapy (Table 3). All patients received dabigatran (110 mg) twice per day and none received low-dose dabigatran. While in the rivaroxaban group, 223 (56.7%) patients received low-dose rivaroxaban (5 mg/day, *n* = 8; 10 mg/day, *n* = 215) (Table 3). Older age was associated with low doses of rivaroxaban (*p* < 0.001) (Table 3). A history of HT and kidney failure was higher in patients who received low-dose rivaroxaban than in those who did not (*p* < 0.001) (Table 3). Patients with high CHA_2_DS_2_-VASc scores and high HAS-BLED scores tended to receive lower doses of rivaroxaban (*p* < 0.001 and *p* = 0.003*, respectively) (Table 3). After PS matching, in the Cox proportional hazards regression analysis, the composite vascular events had no difference between standard and low dose of rivaroxaban groups (HR 0.96 [95% CI 0.5–1.83]; *p* = 0.9) (Table 4; Figure 2). No significant difference was found for ischemic stroke risk between the 2 groups (HR 1.1 [95% CI 0.52–2.41]; *p* = 0.77) (Table 4; Figure 2). There was also no difference between these 2 groups in terms of major bleeding events (HR 1.49 [95% CI 0.33–6.68]; *p* = 0.59) and death rate (HR 0.02 [95% CI 0–8.04]; *p* = 0.19) (Table 4; Figure 2).

**Table 3:** Baseline characteristics of patients received different dose of rivaroxaban.

Characteristics	Low dose (*n* = 223)	Standard dose (*n* = 170)	*p* value
Age, median, [IQR]	78 [70, 83]	73 [67, 77]	<0.001*
Female, *n* (%)	73 (32.7)	41 (24.1)	0.24
Hypertention, *n* (%)	183 (82.1)	68 (40)	<0.001*
Diabetes mellitus, *n* (%)	20 (9.0)	20 (11.8)	0.68
Dyslipidemia, *n* (%)	28 (12.6)	18 (10.6)	0.75
Coronary heart disease, *n* (%)	21 (9.4)	16 (9.4)	0.30
Previous stroke/TIA, *n* (%)	47 (21.1)	38 (22.4)	0.73
History of AF	95 (42.6)	80 (47.1)	0.83
Congestive heart failure, *n* (%)	66 (29.6)	27 (15.9)	0.001*
Kidney failure, *n* (%)	118 (52.9)	51 (30)	<0.001*
Peripheral artery disease, *n* (%)	17 (7.6)	12 (7.1)	0.71
NIHSS median, [IQR]	4 [1, 9]	3 [1, 6]	0.07
Systolic pressure median, [IQR]	146 [131, 165]	144 [129, 161]	0.19
IV Thrombolysis, *n* (%)	35 (15.7)	25 (14.7)	0.97
EVT, *n* (%)	2 (0.9)	4 (2.3)	0.50
Hemorrhage transformation	18 (8.1)	10 (5.9)	0.23
TOAST classification			
Atherosclerosis, *n* (%)	21 (9.4)	17 (10)	0.61
Cardioembolic, *n* (%)	139 (62.3)	101 (59.4)	0.58
Lacunar, *n* (%)	3 (1.3)	1 (0.6)	0.80
Other, *n* (%)	59 (26.5)	51 (30)	0.07
CHA2DS2-VASc>2, *n* (%)	173 (77.6)	106 (62.4)	<0.001*
HAS-BLED, mean (SD)	2.3 (1.1)	2.0 (1.1)	0.003^*^
Hospitalization day, median, [IQR]	9 [7, 11.2]	9.0 [7.0, 12]	0.19
Follow up time (month), median, [IQR]	12 [6, 30]	12 [9, 30]	0.31
Composite vascular (%)	49 (22.0)	22 (12.9)	0.12
Ischemic stroke recurrence (%)	26 (11.7)	17 (10)	0.95
Major bleeding (%)	5 (2.2)	5 (2.9)	0.80

TIA, transient ischemic attack; AF, atrial fibrillation; NIHSS, National Institute of Health stroke scale; IV, Thrombolysis Intravenous thrombolysis; EVT, endovascular treatment; TOAST, Trial of Org 10172 in Acute Stroke Treatment; *p < 0.05.

**Table 4:** Primary and secondary outcomes of standard and reduced dose of rivaroxaban after PS matching.

Outcomes	No. of events	Unadjusted HR (95% CI)	*p* value	Adjusted HR (95% CI)	*p* value
Composite vascular	37 (15.3)				
Reduced	20 (16.5)	1 (Ref)	1 (Ref)	1 (Ref)	1 (Ref)
Standard	17 (14.0)	0.96 (0.50–1.83)	0.90	0.92 (0.48–1.76)	0.92
Ischemic stroke recurrence	26 (10.7)				
Reduced	13 (10.7)	1 (Ref)	1 (Ref)	1 (Ref)	1 (Ref)
Standard	13 (10.7)	1.1 (0.52–2.41)	0.77	1.1 (0.48–2.27)	0.91
Major bleeding	7 (2.9)				
Reduced	3 (2.5)	1 (Ref)	1 (Ref)	1 (Ref)	1 (Ref)
Standard	4 (3.3)	1.49 (0.33–6.68)	0.59	1.49 (0.33–6.68)	0.60
Death	4 (1.7)				
Reduced	4 (3.3)	1 (Ref)	1 (Ref)	1 (Ref)	1 (Ref)
Standard	0	0.02 (0–8.04)	0.19	<0.001 (0–9.99)	0.95

PS, propensity score; Adjusted for age, history of hypertension, congestive heart failure, kidney failure, CHA2DS2-VASc and HAS-BLED score.

## 4 Discussion

The primary findings of the present study are as follows. First, among patients with AIS with NVAF and atherosclerotic narrowing (≥ 2 etiologies), monotherapy with OAC was the most common treatment, followed by APT monotherapy. Combination treatment with APT and OAC was used in only 4.3% of patients in this cohort. Second, in the OAC group, direct OACs were used in most patients, whereas < 1% of patients were prescribed warfarin. Third, low-dose rivaroxaban was prescribed to 56.7% of patients with old age, kidney failure, high CHA_2_DS_2_-VASc and high HAS-BLED scores. Fourth, more composite vascular events and deaths were observed in APT group than that in OAC group, whereas recurrence of ischemic stroke and major bleeding were not significantly different among the 3 groups.

In our investigation, treatment rates for OAC, APT, and combination OAC + APT were 70.7, 25.0, and 4.3%, respectively, compared with 47.0, 19.6, and 33.4%, respectively, in a previous study (19). Our OAC treatment rate was higher, while the combination treatment rate was lower, while the APT was similar. Except for the baseline variables of these 2 cohorts, the different therapeutic options may be due to the lack of guidelines for such situations with ≥ 2 etiologies of AIS and NVAF. Although it has been repeatedly confirmed that NVAF is the most important risk factor for cardioembolic stroke, ischemic stroke―despite the use of OACs―still occurrs in 1–2% of patients with NVAF in pivotal randomized controlled trials, and up to 30% of patients with NVAF who develop ischemic stroke are taking OACs at stroke onset (3, 20–22). Advanced age, HT, DM, renal dysfunction, and other vascular risk factors are common confounders for either NVAF or LAA and SVD. As such, patients with cerebrovascular events experienced stroke due to causes other than cardioembolism, such as LAA and SVD. Ischemic stroke in patients with NVAF is not exclusively cardiogenic in nature.

For patients with AIS with NVAF and LAA, the exact mechanism of stroke events and the presence of cortex-involved lesions with territorial distribution or confluent lesion (> 15mm) with additional lesions involving multiple vascular territories cannot be determined (23–25). Regarding biomarkers for ischemic stroke evaluation with NVAF, the CHA_2_DS_2_-VASc score is recommended by the most influential guidelines as the primary means of stratifying patients (26–28). However the CHA_2_DS_2_-VASc score ignores several potential clinical risk factors for AIS, such as hyperlipidemia and AF type, and has been confirmed to have limited predictive ability for stroke events (16, 29). In our study, due to the co-existence of NVAF and LAA, one-third of patients were diagnosed with an unknown type of AIS according to the TOAST classification. The uncertainty with the mechanism of stroke is an important factor that leads to variability in therapeutic decisions.

In our study, the combination of OAC and APT therapy tended to be prescribed to patients with a history of CAD. In patients with AF who required percutaneous coronary intervention(s) due to acute coronary syndrome, a combination of APT therapy using a P2Y12 inhibitor and OAC yielded a non-inferior number of ischemic events with fewer bleeding complications than triple antithrombotic therapy (dual APT therapy plus OAC) (2, 30, 31). However, caution should be exercised when extrapolating such results to ischemic stroke because it has been suggested that adjunct ATP therapy with OAC does not reduce―or even worsens―the risk for major cardiovascular events (32–34).

In the present study, composite vascular events and mortality were higher in the APT group than those in the OAC group. Because the composite vascular events included death and other vascular events, we suggest that the high composite vascular events recorded in the APT group mainly resulted from the high mortality rate in this group. Older age and more history of CAD may have contributed to the higher mortality in this group.

The present study had several limitations, the first of which was its retrospective design and the use of registry data. Therefore, the possibility of residual confounding factors, as well as uneven patient numbers in various treatment groups, cannot be ruled out. Second, this study analyzed the relationship between discharge treatment and outcomes after 1 year. Drug adherence and whether patients were maintained on the same antithrombotic regimen during the 1 year were unknown. Third, clinical events were captured up to only 1 year after the index stroke, which may have been insufficient and not fully reflect the sustained benefits or risks of treatment over time. However, the follow-up duration for comparing the effectiveness of antithrombotic regimens in most clinical trials is 3 to 12 months. Forth, the discrepancy in numbers as in the combined APT and OAC group only <5% patients were there limiting drawing of relevant conclusions. Fifth, historical imaging limitations such as low resolution or missing sequences may have affected classification accuracy.

According to 2021 AHA/ASA (20) and 2020 ESC guidelines (28), OACs and APT are standard for AF - related and atherosclerotic strokes respectively, but strategies for strokes with both AF and atherosclerosis are unclear. Our study found OAC monotherapy better than APT, potentially attributable to AF-driven cardioembolism dominating clinical phenotypes and pleiotropic OAC effects. Moreover, guideline - advised caution on combined OAC and APT use is echoed by our data showing no benefit of such combination for this stroke subtype.

While our real-world findings provide clinically relevant insights, they require validation through multicenter randomized controlled trials (RCTs) to address unmeasured confounding and generalize across diverse populations. Future multi-center studies will employ longer follow-up time, standardized protocols, stricter inclusion criteria, and harmonized data collection to minimize bias.

In conclusion, results of the present study demonstrated that OAC may reduce the risk for composite outcomes, including recurrent ischemic stroke, ICH, MI, and all-cause mortality, without significant differences in recurrent ischemic stroke 1 year after the index ischemic stroke due to ≥ 2 potential causes associated with AF. Moreover, combination treatment with APT agents and OAC significantly may be associated with a risk for major bleeding without any clinical benefit. Further large-scale clinical trials are required to confirm this association.

## Author’s note

We declare that this article has been published as a PREPRINT (Version 1) available at Research Square [https://doi.org/10.21203/rs.3.rs-4297908/v1].

## Data Availability

The data analyzed in this study is subject to the following licenses/restrictions: the containing information could compromise the privacy of research participants. Requests to access these datasets should be directed to Zheng Zhang, zhangzhengwz@126.com.

## Glossary

AF: Atrial fibrillation
AIS: Acute ischemic stroke
APT: Antiplatelet
ASMD: Absolute mean differences
CAD: Coronary artery disease
CHA2DS2-Vasc: 2 points for history of stroke or age >75 years and 1 point each for congestive heart failure, hypertension, diabetes mellitus, vascular disease, age between 65 and 74 years, and female sex
DL: Dyslipidemia
DM: Diabetes mellitus
EVT: Endovascular recanalization therapy
GI: Gastrointestinal bleeding
HAS-BLED: Hypertension, abnormal renal/liver function, stroke, bleeding history or predisposition, labile INR, elderly, drugs/alcohol concomitantly
HT: Hypertension
ICH: Intracerebral hemorrhage
IV: Intravenous
LAA: Large artery atherosclerosis
MI: Myocardial infarction
NIHSS: National Institutes of Health Stroke Scale score
NVAF: Non-valvular atrial fibrillation
OAC: Oral anticoagulants
PS: Propensity score
SD: Standard deviations
SVD: Small-vessel disease
HR: Hazard ratio
TOAST: Trial of Org 10172 in Acute Stroke Treatment
