# Supplementary material for: Antithrombotic agents and effect on outcomes in ischemic stroke with atrial fibrillation and large artery atherosclerosis: a real-world study
Source: Front Neurol. 2025 Jun 9;16:1576250. doi: 10.3389/fneur.2025.1576250 (PMC12183024; doi:10.3389/fneur.2025.1576250)
Supplement: Supplementary file 1 [file Table_1.DOCX]

| **ASMD of baseline characteristics of ATP and OAC treatment groups** | | | | | |
| --- | --- | --- | --- | --- | --- |
|  | **Antiplatelet**  **(n=140)** | **ASMD** | **Anticoagulant**  **(n=140)** | **ASMD** | ***p* value** |
| **Age years median, [IQR]** | 78 [70, 85] | 0.09 | 76.5 [69.8, 84] | 0.1 | 0.23 |
| **Female, n (%)** | 51 (36.7) | 0 | 29/70 (35.7) | 0.07 | 0.49 |
| **Hypertension, n (%)** | 122 (87.4) | 0.02 | 57/70 (80.5) | 0.014 | 0.35 |
| **Diabetes mellitus, n (%)** | 20(14.3) | 0.002 | 16/70 (8.9) | 0.037 | 0.19 |
| **Dyslipidemia, n (%)** | 22 (15.7) | 0.003 | 31 (6.9) | 0.002 | 0.43 |
| **Coronary heart disease, n (%)** | 18 (12.9) | 0.0002 | 8/70 (9.6) | 0.09 | 0.79 |
| **History of AF** | 54 (38.6) | 0.013 | 30/70 (44.5) | 0.0002 | 0.61 |
| **Congestive heart failure, n (%)** | 40 (28.6) | 0.001 | 18/70(23.0) | 0.03 | 0.7 |
| **Previous stroke/TIA, n (%)** | 32 (22.9) | 0.003 | 18/70 (21.9) | 0.04 | 0.69 |
| **Kidney failure, n (%)** | 70 (50) |  | 37/707 (40.9) | 0.02 | 0.74 |
| **Peripheral artery disease, n (%)** | 10 (7.1) | 0.23 | 6/70(6.9) | 0.003 | 0.75 |
| **Smoking, n (%)** | 28 (20) | 0.004 | 17 (31) |  | 0.54 |
| **NIHSS median, [IQR]** | 4.0 [1,10] | 0.02 | 3.0 [1,3] | 0.02 | 0.67 |
| **IV Thrombolysis, n (%)** | 18 (12.9) | 0.07 | 9 (14.7) | 0.06 | 1 |
| **EVT, n (%)** | 2 (1.4) | 0.01 | 0 (1.3) | 0.05 | 1 |
| **Hemorrhage transformation** | 30 (21.4) | 0.007 | 9 (6.4) | 0.005 | 0.18 |
| **GI** | 2 (1.4) | 0.004 | 4 (1.3) | 0.03 | 0.17 |
| **CHA_2_DS_2_-VASc>2, n (%)** | 118 (84.8) | 0.055 | 58 (69.8) | 0.13 | 0.82 |
| **HAS-BLED, mean (SD)** | 1.8 (1.2) | 0.08 | 2.2 (1.1) | 0.03 | 0.86 |
| **Hospitalization day, median, [IQR]** | 9.0 [6,12] | 0.001 | 9.0 [7,12] | 0.001 | 0.98 |

ASMD absolute mean difference; APT antiplatelet; OAC oral anticoagulant; AF atrial fibrillation; TIA transient ischemic attack; Kidney failure creatinine clearance <50ml/min; NIHSS National Institute of Health stroke scale; IV Thrombolysis Intravenous thrombolysis; EVT endovascular treatment; GI gastrointestinal bleeding;
